# Analysis of Risk Factors and Establishment of a Prediction Model for Endoscopic Primary Bile Reflux: A Single-Center Retrospective Study

**DOI:** 10.3389/fmed.2021.758771

**Published:** 2021-11-10

**Authors:** Li Chen, Guoying Zhu, Ling She, Yongnian Ding, Changqing Yang, Fengshang Zhu

**Affiliations:** ^1^Department of Gastroenterology, School of Medicine, Tongji Hospital, Tongji University, Shanghai, China; ^2^State Key Laboratory of Pathogenesis, Prevention, and Treatment of High Incidence Diseases in Central Asia, Xinjiang Medical University, Ürümqi, China; ^3^Department of Gastroenterology, Second Affiliated Hospital of Xinjiang Medical University, Ürümqi, China; ^4^Department of Clinical Nutrition, School of Medicine, Putuo People's Hospital, Tongji University, Shanghai, China

**Keywords:** primary bile reflux, endoscope, risk factors, prediction model, retrospective study

## Abstract

**Background:** Endoscopic primary bile reflux is one of the main diagnostic criteria for bile reflux gastritis (BRG). Presently, the risk factors and prediction models of endoscopic primary bile reflux (EPBR) in gastropathy patients who cannot or will not undergo endoscopy due to contraindications are not clear. Thus, this study aimed to evaluate the risk factors of EPBR and to establish and verify a prediction model.

**Methods:** A series of 844 patients (564 subjects with EPBR and 280 control subjects) were retrospectively selected for this study and divided into a training set (*n* = 591) and a validation set (*n* = 253) according to the usual ratio of 70:30% for the subsequent internal validation of the logistic regression model for EPBR. Fifteen parameters that might affect the occurrence of EPBR were collected. Subsequently, univariate and stepwise logistic regression analyses were introduced to reveal the risk factors and the multivariate prediction model. An R package was dedicated to the corresponding internal validation of the EPBR model.

**Results:** The univariate analysis showed that gender, age, smoking, Helicobacter pylori (*H. pylori*) infection status, metabolic syndrome (MS), non-steroidal anti-inflammatory drugs (NSAIDs) use history, and previous medical histories of chronic liver diseases, cholelithiasis, and erosive gastritis were statistically significant between the two groups (*P* < 0.05). Multivariate regression described that being a male [OR (95%confidence interval (CI)) = 2.29 (1.50–3.50), *P* < 0.001], age≥45 years old [OR (95% CI) = 4.24 (2.59–6.96), *P* < 0.001], *H. pylori* infection status [OR (95% CI) = 2.34 (1.37–4.01), *P* = 0.002], MS [OR (95% CI) = 3.14 (1.77–5.54), *P* < 0.001], NSAIDs use history [OR (95% CI) = 1.87 (1.03–3.40), *P* = 0.04], cholelithiasis history [OR (95% CI) = 3.95 (2.18–7.18), *P* < 0.001] and erosive gastritis history [OR (95% CI) = 6.77 (3.73–12.29), *P* < 0.001] were the risk factors for the occurrence of EPBR. Based on the results of these risk factors, an EPBR prediction model with an adequate calibration and excellent discrimination was established [area under the curve (AUC): 0.839, 95% CI = 0.806–0.872].

**Conclusions:** Being a male, age ≥ 45 years old, *H. pylori* infection, histories of MS, NSAIDs use, cholelithiasis, and erosive gastritis appear to be the risk factors for EPBR, and our favorable prediction model might be an option for the prediction of EPBR.

## Introduction

Bile reflux gastritis, also known as alkaline reflux gastritis (ARG), refers to the chronic inflammation, erosion, and even ulceration in the gastric mucosa caused by excessive duodenal fluid (including bile, pancreatic, and intestinal fluid) refluxing into the stomach (1). The action component of duodenal reflux fluid is bile acid, which has an accumulative damaging effect on the gastric mucosal barrier and can induce chronic inflammation, erosion, ulcers, gastroesophageal reflux, and even carcinogenesis (2–4). As a common digestive disease, bile reflux gastritis (BRG) accounts for about 22.6% of chronic gastritis (5). Bile reflux gastritis that originated in a non-operative stomach is referred to as primary bile reflux gastritis (PBRG), while BRG that occurred after gastric pylorus surgery is called secondary bile reflux gastritis (SBRG). Endoscopic primary bile reflux is part of the most important diagnostic criteria for PBRG (6, 7). Long-term endoscopic primary bile reflux (EPBR) may also lead to the hyperplasia of gastric epithelial pits and esophageal squamous epithelium, and may even be associated with intestinal metaplasia or cancer (8). However, to date, the etiologies and risk factors of EPBR are not well-understood, especially for gastropathy patients who cannot or will not undergo further endoscopy due to contraindications.

Past two conflicting studies have explored the influence of psychological factors and *H. pylori* infection on EPBR (4, 5). Another study revealed that EPBR might be involved with sex, age, and fasting time (9). These findings provided a preliminary basis for further research. Our study aimed to further elucidate more possible factors related to the occurrence of EPBR, and eventually, to design a prediction model that provides a valuable evaluation tool for patients with EPBR. The significance of this study is to offer clues for clinical empirical diagnosis and treatment.

## Materials and Methods

### Study Design and Participants

**Figure 1:**
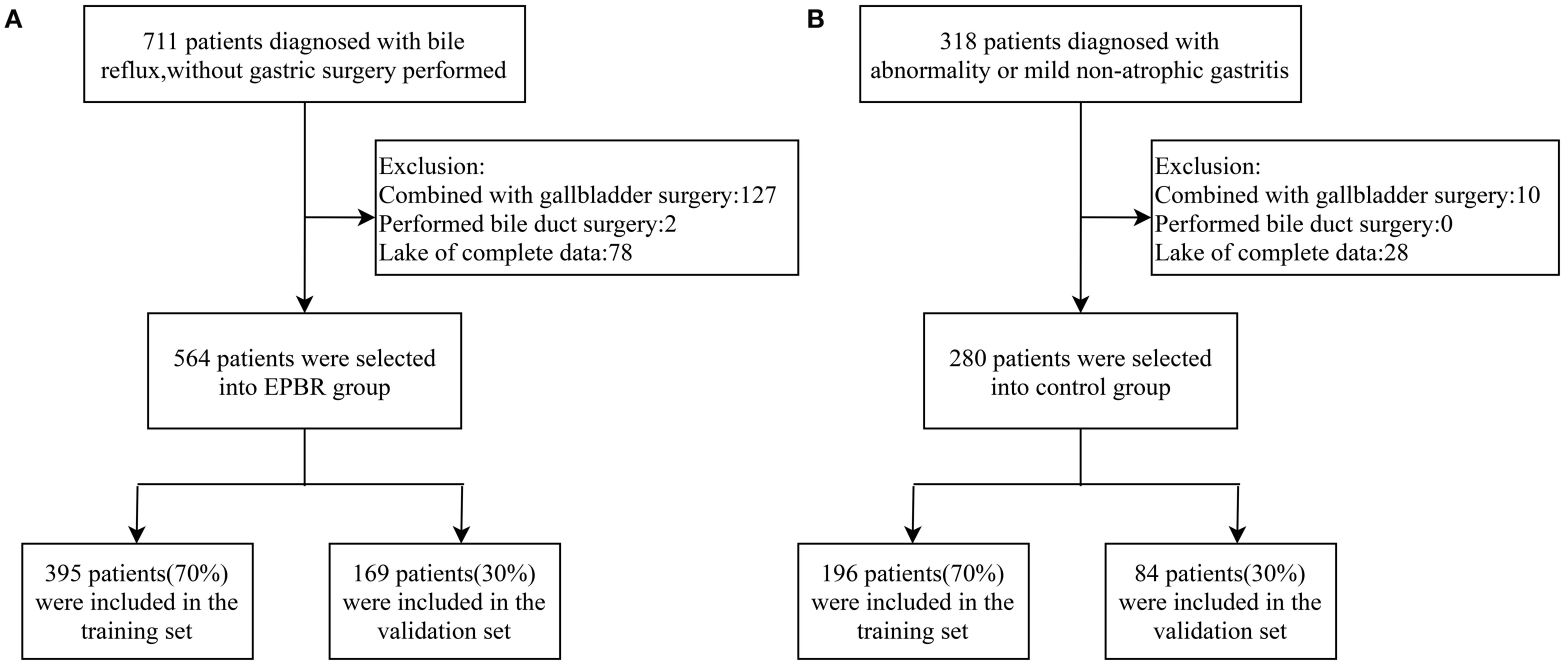
Schematic diagram of screening process for primary bile reflux group **(A)** and control group **(B)**.

A total of 1,029 patients admitted to the Tongji Hospital of Tongji University from January 2017 to December 2020 were assigned the analytical data, including 711 subjects with EPBR and 318 control subjects. The studies involving human participants were reviewed and approved by the Ethics Committee of Tongji Hospital, School of Medicine, Tongji University. The patients or participants involved in this study had provided their written informed consent. Three endoscopists performed gastroscopies, and all of them were skilled in endoscopic procedures and had the same diagnostic criteria for EPBR. All the patients were excluded from gastric surgery and had not taken proton pump inhibitors (PPI) or ursodeoxycholic acid in 7–10 days prior to endoscopy. The same exclusion criteria in the two groups were applied to one of the following: patients with histories of gastrectomy, cholecystectomy, other biliary surgery (they are generally considered as predisposing factors for SBRG), and incomplete clinical medical data (Figures 1A,B). Consequently, a series of 844 patients with complete medical records were retrospectively divided into the EPBR group (*n* = 564) and the control group (*n* = 280) (Figures 1A,B). The control group means no abnormality or only mild non-atrophic gastritis under the gastroscope. For the subsequent internal validation of the logistic regression model for EPBR, the two groups were successively selected to build the training set and the validation set according to the usual ratio of 70%:30% (training set: *n* = 591, validation set: *n* = 253) (Figures 1A,B).

The patients with EPBR were enrolled as endoscopically confirmed. The determination of excessive EPBR in the stomach under endoscopy was mainly based on the mucus lake bile staining when the endoscope is entered into the gastric cavity (10). According to the color of the mucus lake, the EPBR severity was classified into four levels: mucus lake is clear and transparent (Grade 0); mucus lake is clear and light yellow (Grade I); mucus lake is yellow and clear (Grade II); the mucous lake is pale yellow or dark green (Grade III) (6, 11). The EPBR of grade 0–I, also called physiological reflux, is unlikely to cause digestive symptoms and pathological gastric mucosal lesions. Instead, EPBR of grade II–III may induce upper gastrointestinal symptoms and pathological gastric mucosal lesions, which is considered as pathological reflux (12).

### Clinical Variables

In the present study, the succeeding 15 parameters that might influence the occurrence of EPBR were gathered after obtaining the informed consent of the patients. Fifteen parameters that might affect the occurrence of EPBR, namely, gender, age, body mass index (BMI), smoking, alcohol consumption, Helicobacter pylori (*H.pylori*) infection status, metabolic syndrome (MS), non-steroidal anti-inflammatory drugs (NSAIDs) use history, psychological factors, allergic constitution, gastrointestinal symptoms, and previous medical histories of chronic liver diseases, cholelithiasis, erosive gastritis, and pancreatic diseases were collected.

The medical history of psychotropic medication of the patient and the anxiety and depression scale was used as the criteria to assess whether the patient had mental health factors. The diagnostic criteria for MS proposed by the International Diabetes Federation (IDF) in 2005 were adopted (13). The specific diagnostic definitions for MS were as follows: central obesity (waist circumference as the tangent point, male ≥ 90 cm, female≥80 cm) and any two of the following four indicators: (1) raised triglycerides (TG)>1.7 mmol/L, or specific treatment for this lipid abnormality; (2) reduced HDL-cholesterol (HDL-C): <1.03 mmol/L in men and <1.29 mmol/L in women, or being correspondingly treated; (3) raised blood pressure: ≥130/85 mmHg, or treatment of previously diagnosed hypertension; (4) raised fasting plasma glucose (FPG): ≥5.6 mmol/L or previously diagnosed type 2 diabetes. The gastrointestinal symptoms referred to abdominal pain, abdominal distension, heartburn, bitter taste, or vomiting bile before endoscopy, with at least one of which was regarded as having gastrointestinal symptoms. Moreover, the eligible contents of chronic liver diseases were viral hepatitis, fatty liver, cirrhosis, and liver malignancy. Cholelithiasis meant cholecystitis, gallstones, and malignant tumors of the biliary tract. The patients with pancreatic diseases included acute or chronic pancreatitis and pancreatic tumors. The selection principle of the 14 potential risk factors above for inducing or deteriorating EPBR mainly hinged on previous literature and our clinical practice experience (14–18).

### Statistical Analysis

The statistical analysis was conducted with SPSS version 20.0 (IBM Corp., Armonk, New York, United States) and R software (version 4.0.2; http://www.Rproject.org) using an alpha level of 0.05. The quantitative data with normal distribution were calculated for the mean with SDs, and the quantitative data with abnormal distribution were expressed as median with interquartile ranges, whereas the frequencies were determined by the categorical values. The Chi-square test or *Fisher's* exact probability method, the Student *t-*test, and *Wilcoxon* rank-sum test was employed for the analysis of the categorical, continuous, and ordinal variables between the groups, respectively. The predictors of the variables were tested in univariate and multivariate logistic regression analyses for their association with EPBR. The discriminatory ability of the logistic regression model was quantified using the receiver operating characteristic (ROC) curve and the area under the curve (AUC). The calibration of the nomogram was performed by plotting the observed outcome probabilities. The Hosmer–Lemeshow (H–L) test was employed to evaluate how well the percentage of the observed probability matched the percentage of the predicted probability. A *P* < 0.05 was considered statistically significant.

## Results

### Description of the Subjects

According to the inclusion and exclusion criteria, 564 subjects in the reflux group and 280 subjects in the control group were screened, respectively (Figures 1A,B). The training set used for establishing the logistic regression model consisted of 395 subjects from the reflux group and 196 subjects from the control group. Meanwhile, 169 subjects from the reflux group and 84 subjects from the control group constituted the validation set and were assigned to the internal validation of the EPBR prediction model (Figures 1A,B). The equilibrium test showed that there was no system selection bias between the two data sets (Supplementary Table 1). Therefore, it is reasonable and feasible to establish a logistic regression model through the above data sets.

### Univariate Analysis

Our univariate analysis studies revealed that there were statistically significant differences in gender, age, smoking, *H. pylori* infection status, MS, histories of NSAIDs use, chronic liver diseases, cholelithiasis, and erosive gastritis between the two groups (*P* < 0.05) (Table 1).

**Table 1:** Univariate analysis of the risk factors for bile reflux in the training set.

**Factor**	**Total (*n* = 591)**	**Bile reflux**		***P-*value**
		**No (*n* = 196)**	**Yes (*n* = 395)**	
**Gender, n (%)**				<0.001
Female	296 (50.08)	127 (64.80)	169 (42.78)	
Male	295 (49.92)	69 (35.20)	226 (57.22)	
Age, Mean ± SD	50.18 ± 13.72	43.27 ± 13.60	53.62 ± 12.44	<0.001
**Age, n (%)**				<0.001
<45	194 (32.83)	110 (56.12)	84 (21.27)	
45–59	201 (34.01)	51 (26.02)	150 (37.97)	
≥60	196 (33.16)	35 (17.86)	161 (40.76)	
Height, M (Q_1_, Q_3_)	1.65	1.66	1.65	0.367
	(1.60, 1.72)	(1.60, 1.73)	(1.60, 1.71)	
Weight, Mean ± SD	64.23 ± 13.45	65.04 ± 15.85	63.82 ± 12.08	0.346
BMI, Mean ± SD	23.14 ± 3.87	23.28 ± 4.47	23.07 ± 3.54	0.579
**BMI, n (%)**				0.625
<18.5	59 (9.98)	20 (10.20)	39 (9.87)	
18.5–23.9	300 (50.76)	102 (52.04)	198 (50.13)	
≥24	232 (39.26)	74 (37.76)	158 (40.00)	
**NSAIDs use history, n (%)**				0.005
No	491 (83.08)	175 (89.29)	316 (80.00)	
Yes	100 (16.92)	21 (10.71)	79 (20.00)	
**Chronic liver diseases, n (%)**				0.002
No	521 (88.16)	184 (93.88)	337 (85.32)	
Yes	70 (11.84)	12 (6.12)	58 (14.68)	
**Cholelithiasis, n (%)**				<0.001
No	447 (75.63)	176 (89.80)	271 (68.61)	
Yes	144 (24.37)	20 (10.20)	124 (31.39)	
***H. pylori infection status, n (%)***				<0.001
No	446 (75.47)	166 (84.69)	280 (70.89)	
Yes	145 (24.53)	30 (15.31)	115 (29.11)	
**Psychological factors, n (%)**				0.101
No	408 (69.04)	144 (73.47)	264 (66.84)	
Yes	183 (30.96)	52 (26.53)	131 (33.16)	
**Allergic constitution, n (%)**				0.075
No	473 (80.03)	165 (84.18)	308 (77.97)	
Yes	118 (19.97)	31 (15.82)	87 (22.03)	
**Erosive gastritis, n (%)**				<0.001
No	421 (71.24)	178 (90.82)	243 (61.52)	
Yes	170 (28.76)	18 (9.18)	152 (38.48)	
**Smoking, n (%)**				0.011
No	478 (80.88)	170 (86.73)	308 (77.97)	
Yes	113 (19.12)	26 (13.27)	87 (22.03)	
**Alcohol consumption, n (%)**				0.259
No	531 (89.85)	180 (91.84)	351 (88.86)	
Yes	60 (10.15)	16 (8.16)	44 (11.14)	
**Metabolic syndrome, n (%)**				<0.001
No	460 (77.83)	171 (87.24)	289 (73.16)	
Yes	131 (22.17)	25 (12.76)	106 (26.84)	
**Pancreatic diseases, n (%)**				0.171
No	562 (95.09)	183 (93.37)	379 (95.95)	
Yes	29 (4.91)	13 (6.63)	16 (4.05)	
**Gastrointestinal symptoms, n (%)**				0.129
No	342 (57.87)	122 (62.24)	220 (55.70)	
Yes	249 (42.13)	74 (37.76)	175 (44.30)	

### Multivariate Analysis

Multivariate regression described that being male [OR (95% confidence interval (CI) = 2.29 (1.50–3.50), *P* < 0.001], age ≥ 45 years old [OR (95% CI) = 4.24 (2.59–6.96), *P* < 0.001], *H. pylori* infection status [OR (95%CI) = 2.34 (1.37–4.01), *P* = 0.002], MS [OR (95% CI) = 3.14 (1.77–5.54), *P* < 0.001], NSAIDs use history [OR (95% CI) = 1.87 (1.03–3.40), *P* = 0.04], cholelithiasis history [OR (95% CI) = 3.95 (2.18–7.18), *P* < 0.001], and erosive gastritis history [OR (95% CI) = 6.77 (3.73–12.29), *P* < 0.001] were the risk factors for the occurrence of EPBR (Table 2).

**Table 2:** Logistic multivariate analyses of risk factors for primary bile reflux in the training set.

**Factor**	**β**	**S.E**	**Wald**	***P***	**OR**	**95% CI**	
						**Lower**	**Upper**
Constant	−1.860	0.241	59.405	<0.001			
Gender (Male)	0.829	0.216	14.723	<0.001	2.291	1.500	3.500
**Age**							
<45					Ref.		
45–59	1.445	0.253	32.729	<0.001	4.243	2.586	6.961
≥60	1.937	0.275	49.477	<0.001	6.935	4.043	11.896
NSAIDs use history (Yes)	0.628	0.307	4.169	0.041	1.874	1.025	3.423
Cholelithiasis (Yes)	1.374	0.304	20.378	<0.001	3.952	2.176	7.176
*H. pylori* infection (Yes)	0.851	0.274	9.643	0.002	2.342	1.369	4.008
Erosive gastritis (Yes)	1.912	0.305	39.410	<0.001	6.766	3.725	12.290
Metabolic syndrome (Yes)	1.143	0.290	15.478	<0.001	3.135	1.774	5.539

### Establishment of the Prediction Model

Based on the results of the multivariate analysis, a formula for predicting the probability of EPBR was computed as follows: P = e^X^/(1+e^X^), X = 0.829X_1_ + 1.445 X_2_ or 1.937X_2_ + 0.628X_3_ + 1.374X_4_ + 0.851X_5_ + 1.912X_6_ + 1.143X_7_-1.860 (Table 2). The values of the various parameters in the formula were different: X_1_ = gender (female = 0, male = 1); X_2_ = age (<45 = 0, 45–59 = 1 for 1.445 X_2_; ≥ 60 = 1 for 1.937 X_2_); X_3_ = NSAIDs use history (no = 0, yes = 1); X_4_ = cholelithiasis history (no = 0, yes = 1); X_5_ = *H. pylori* infection status (no = 0, yes = 1); X_6_ = erosive gastritis history (no = 0, yes = 1); and X_7_ = MS history (no = 0, yes = 1). The cut-off value of the prediction model was 0.667.

**Figure 2:**
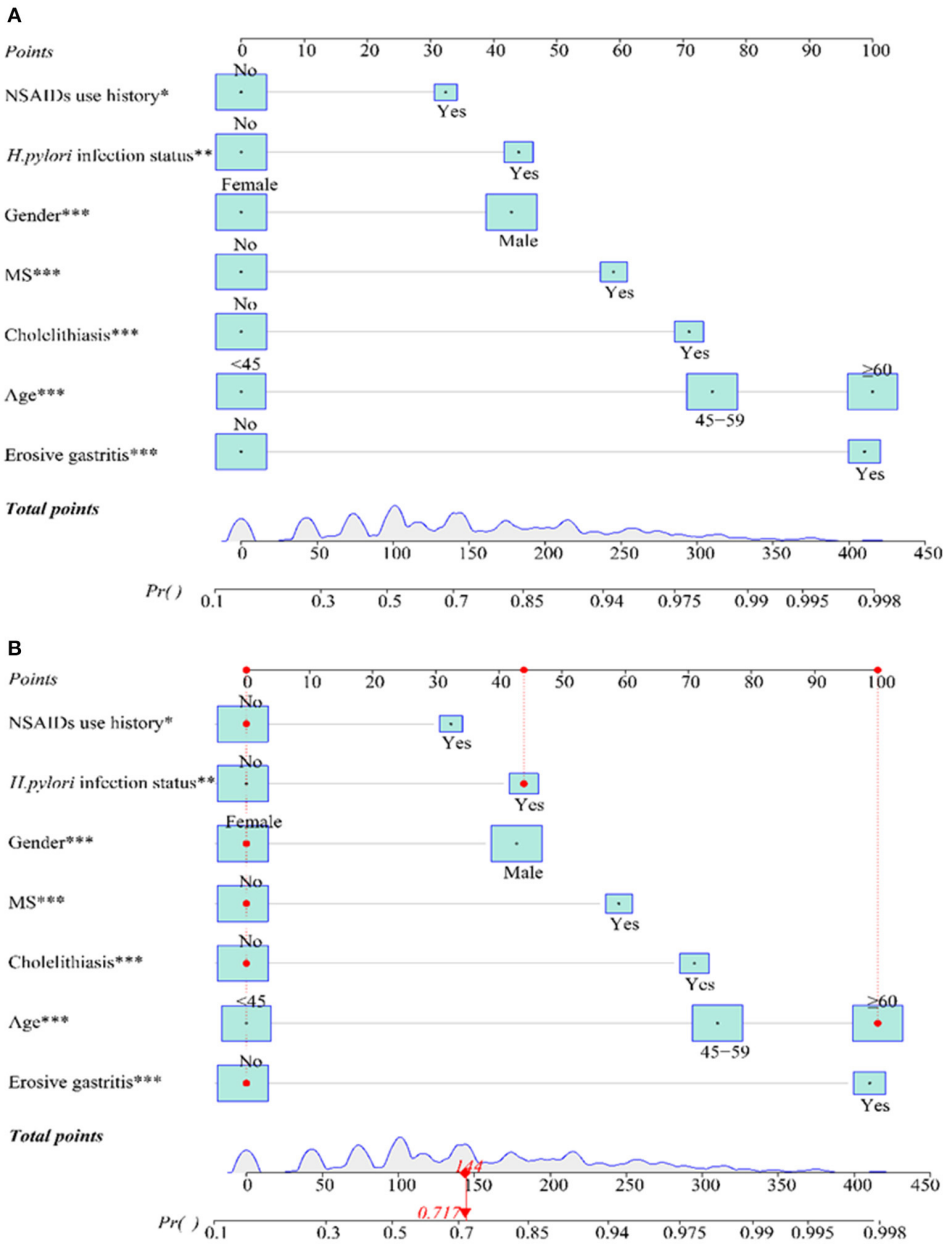
Nomogram of prediction model **(A)** and model test **(B)**.

A nomogram that depicted the multivariate impact of each risk factor was further developed (Figure 2A). A further descriptive explanation and example of the logistic regression model was as follows: we randomly selected a patient from the data, who was with the clinical characteristics of female, age ≥ 60 years old, NSAIDs use history, no cholelithiasis history, and *H. pylori* infection, no erosive gastritis, and MS history. Consequently, the logistic regression model score for this patient was 144 and the corresponding probability of EPBR occurrence was 0.717. Seeing that this score exceeded the cut-off value (0.667), this patient should be identified with a serious probability of EPBR. As expected, the de facto confirmed EPBR in this patient and provided a testament for the accuracy of the internal validation for the prediction model (Figure 2B).

### Prediction Model Evaluation and Internal Validation

**Figure 3:**
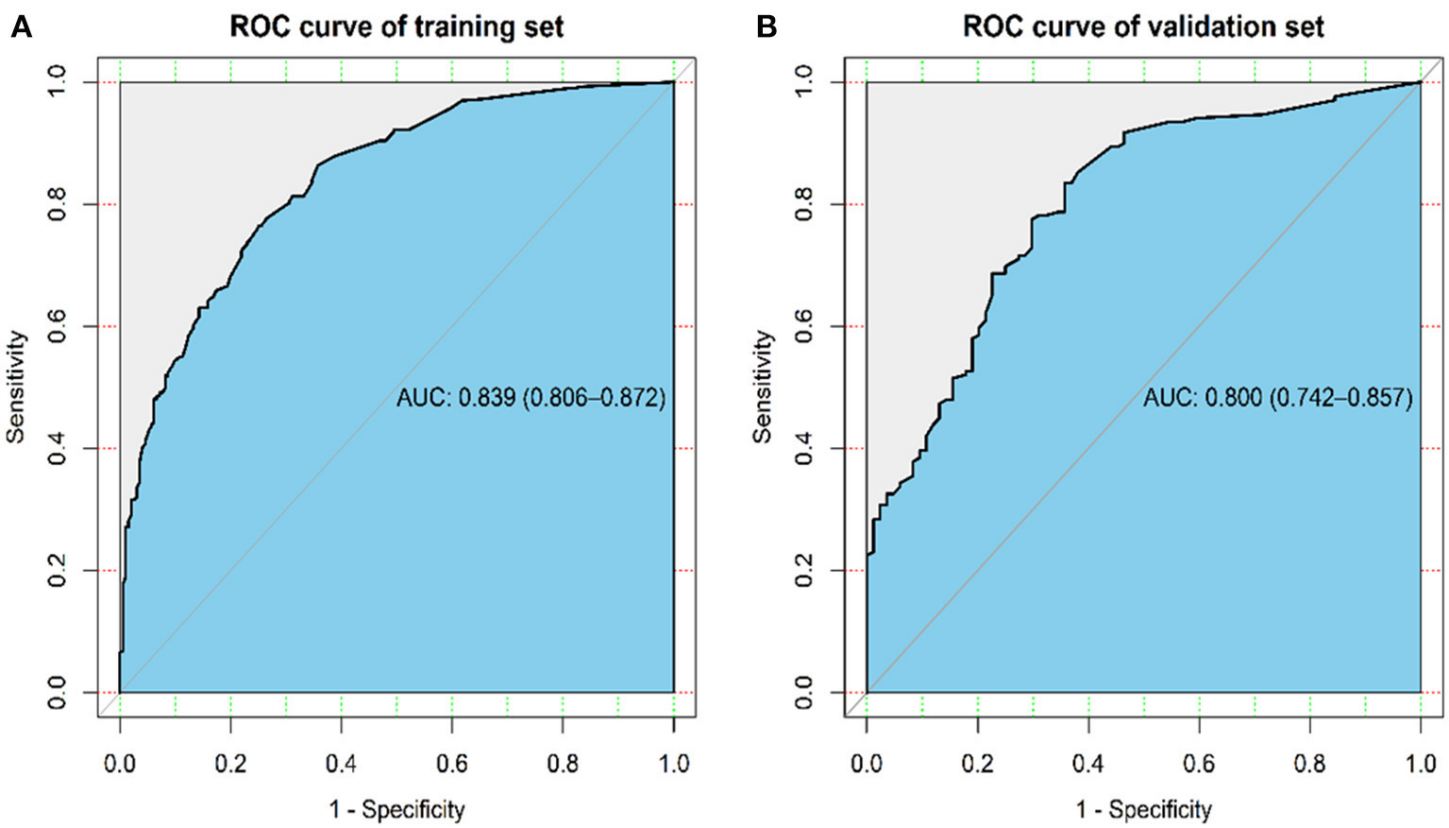
ROC curve and AUC for prediction model in the training **(A)** and validation set **(B)**.

The ROC analysis for the logistic regression model was computed to judge its clinical discrimination. As shown in Figure 3, the ROC analysis revealed that this model had an eminent discrimination ability due to the results of AUC 0.839 (95% CI, 0.806–0.872) in the training set (Figure 3A) and 0.800 (95% CI, 0.742–0.857) in the validation set (Figure 3B), respectively.

**Figure 4:**
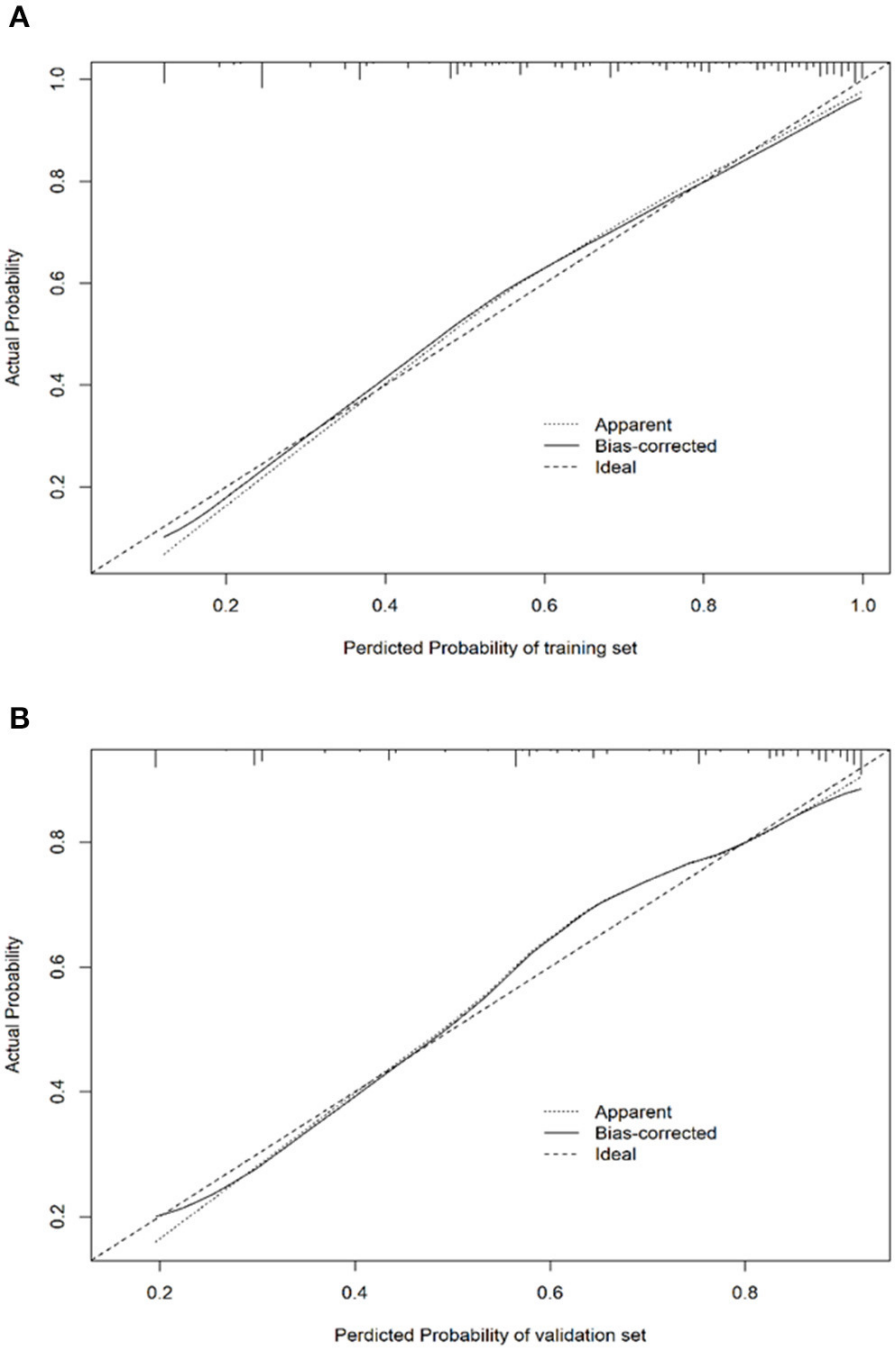
Calibration plots for prediction model in the training set **(A)** and validation set **(B)**.

In addition to these evaluations, the H–L test was adopted for the calibration of the logistic regression model. The calibration plots for the probabilities of EPBR described that the logistic regression model was adequately calibrated, with no indication of systematic under-or overestimation of EPBR rate (Figures 4A,B). As a cut-off value is 0.667, the regression model features, such as the sensitivity, specificity, positive predictive value (PPV), and negative predictive value (NPV), were 0.765 (95% CI = 0.723–0.806), 0.750 (95% CI = 0.689–0.811), 0.860 (95% CI = 0.824–0.897), and 0.612 (95% CI = 0.551–0.674) in the training set and 0.686 (95% CI = 0.616–0.756), 0.774 (95% CI = 0.684–0.863), 0.859 (95%CI = 0.801–0.918), and 0.551(95% CI = 0.461–0.641) invalidation set, respectively (Table 3).

**Table 3:** Features of the prediction model in the training and validation set.

**Index (95% CI)**	**Training set**	**Validation set**
AUC	0.839 (0.806–0.872)	0.800 (0.742–0.857)
Sensitivity	0.765 (0.723–0.806)	0.686 (0.616–0.756)
Specificity	0.750 (0.689–0.811)	0.774 (0.684–0.863)
PPV	0.860 (0.824–0.897)	0.859 (0.801–0.918)

## Discussion

In general, the coordinated movement of the stomach and duodenum rarely causes EPBR. However, when the duodenum becomes antiperistaltic and the pyloric closure is incomplete, in the meantime, bile will flow back into the stomach (19). Accompanied by delayed gastric emptying, bile acids continue to interact with the gastric mucosa and finally result in well-recognized damage (20). Any factor that causes gastrointestinal motility disorder and anatomical abnormality can cause pathologic duodenogastric reflux. Endoscopic primary bile reflux was classified as primary and secondary type (21), depending on whether the patient has a gastrectomy history. In addition to other endoscopic features such as the co-existence that changes mucosa: hyperemia, fragility, and erosions, EPBR is one of the mandatory diagnostic criteria for PBRG (6, 7). For patients who cannot or will not undergo endoscopy due to contraindications, the determination of EPBR is particularly critical for clinical empirical treatment. Furthermore, our clinical experience is not identical to the predictors of EPBR reported in most historical studies. Therefore, the purpose of this study was to summarize the predictors and prediction models of EPBR in a real-world setting with a large sample size.

In this retrospective study, 15 clinical parameters that possessed a strong possibility of predicting EPBR were analyzed, and seven risk factors including being male, age ≥45 years old, *H. pylori* infection, previous medical histories of NSAIDs use, cholelithiasis, erosive gastritis, and MS were accessed for the first time. Furthermore, after validating the regression formula through the ROC and H-L test, the evidence from our retrospective study suggested that the prediction model for EPBR provides a favorable reference for clinical empirical treatment.

As a recognized etiologic agent, *H. pylori* colonizes the gastric mucosa and causes gastritis, peptic ulcers, and gastric cancer (21, 22). Even now, the relationship between EPBR and *H. pylori* infection remains controversial (23). Several studies attempted to address the association of EPBR and *H*. pylori infection with the occurrence and development of chronic gastritis and gastric cancer (9, 14, 24–26). One study implied that bile acid, which continued in gastric juice, had the extraordinary capabilities of promoting ulcer healing and inhibiting the growth of *H. pylori* (27). In contrast, substantial data believed EPBR to be consistently reduced after successful *H. pylori* eradication. The *H. pylori* infection status may affect EPBR by increasing gastrin secretion and altering the duodenal movement (28). This notion meant that the two had a synergistic effect in inducing chronic gastritis (5). A multi-center study of 2,283 subjects from 14 institutions in Japan found that a high concentration of EPBR increased the risk of intestinal metaplasia regardless of the *H. pylori* infection status (29). Partially contrary to the findings of previous literature reports, our results demonstrated that *H. pylori* infection and erosive gastritis history coexisted in our prediction model. It is well-known that the prolonged exposure of gastric mucosa to bile acid and *H. pylori* infection may result in histopathological changes (30, 31). Our study proves to be a likely overlap between *H. pylori*-associated gastritis and BRG. Consequently, the sensitive identification and appropriate treatment of EPBR should be admitted while considering the presence of *H. pylori* infection.

Moreover, a history of NSAIDs use, besides *H. pylori* infection and EPBR, is likewise a common cause of chronic gastritis (32–34). Data from *in vivo* studies has shown that administration of NSAIDs decreased the prostaglandin concentration in the gastric mucosa but did not increase the mucosal damage in *H. pylori*-induced gastritis, which is ascribed to the elevated expression of cyclooxygenase-2 (COX-2) induced by *H. pylori* (8). In our multivariate logistic regression model, *H. pylori* infection and the history of NSAIDs use were incorporated into the prediction model as risk factors. However, the relationships between *H. pylori* infection, NSAIDs use, and EPBR in gastric mucosal damage are intricate, which is yet to be clarified by more basic and clinical researches.

During the past few years, several studies have shown that EPBR is more frequent in elderly male patients, which may be associated with an increased rate of gastrectomy and operation of the biliary tract (35). However, a study reported no distinguishable differences in EPBR occurrence between the younger (median age 25 years) and older (median age 51 years) healthy volunteers (36). Another related study believed that the rate of EPBR in women and middle-aged patients was higher than in men and in young and elderly patients (9). Here we demonstrated that EPBR was more common in male patients aged 45 years or older. An appropriate interpretation of the above conclusions imputes to population characteristics or genetic background and synchronously implies that a multi-center prospective study with a larger sample size is needed.

It is currently generally accepted that bile reflux is frequently disclosed in patients with chronic calculous cholecystitis patients (67–80%) and cholecystectomy (89%) (11, 37). Previous studies have evaluated that the predisposition toward EPBR in patients with cholelithiasis can probably be associated with changes in the gut hormone induced by biliary tract disease (12, 38, 39). There was no clear evidence to suggest that EPBR is clinically related to MS patients in previous studies. Meanwhile, in our clinical practice, we realized that EPBR patients are often accompanied by MS. Our results go beyond the previous reports, showing that MS is also a risk factor for EPBR. Existing studies have emphasized that the occurrence of MS is always related to age and gender (40–43). These results underscored the idea that the prevalence of MS tended to be higher with age in women than in men, driven primarily by an increase in abdominal obesity and a decrease in the HDL-C levels (44). Although, whether MS interacts with age, gender, and other factors to play an essential role in EPBR remains undefined. At present, the prevalence of MS continues to rise globally (45), and the frequency of cardio-cerebrovascular events is concerned with MS. Here we demonstrated that EPBR requires more attention instead of just the frequency of cardio-cerebrovascular events associated with MS. In the future, it will be necessary to explore the relationship between MS and EPBR after excluding the other risk factors.

The limitations of the present studies naturally include only a single-center retrospective experience, and more clinical data from other medical institutions for external validation are urgently needed. Meanwhile, gastric cancer may be misdiagnosed as acid reflux, leading to certain false positives in our study, which needs to be paid attention to. Moreover, the diagnosis of EPBR is just configured based on endoscopy without 24 h gastric bilirubin monitoring, and its causal relationship, as well as underlying mechanisms, should be further confirmed through prospective and in-depth studies.

In conclusion, being male, age ≥ 45 years old, *H. pylori* infection, histories of MS, NSAIDs use, cholelithiasis, and erosive gastritis appear to be the risk factors for EPBR. Meanwhile, this favorable prediction model might be an option for the prediction of EPBR.

## Data Availability Statement

The original contributions presented in the study are included in the article/Supplementary Material, further inquiries can be directed to the corresponding author/s.

## Ethics Statement

The studies involving human participants were reviewed and approved by Ethics Committee of Tongji Hospital, School of Medicine, Tongji University. The patients/participants provided their written informed consent to participate in this study.

## Author Contributions

LC and GZ acquired and analyzed the data and participated in drafting the manuscript. LS, YD, FZ, and CY provided comprehensive case data and analyzed the data. FZ contributed to the concept and design of the work, reviewed, and revised the manuscript. All authors contributed to the article and approved the submitted version.

## Funding

This work was supported by the State Key Laboratory of Pathogenesis, Prevention, and Treatment of High Incidence Diseases in Central Asia Fund (No.SKL-HIDCA-2021- DX9, Urumqi, China), Xinjiang Uygur Autonomous Region Special Regional Collaborative Innovation Project (Science and Technology Aid Xinjiang program, No. 2021E02075, China), and the Shanghai Key Specialty Construction Project (ZK2019C16).

## Conflict of Interest

The authors declare that the research was conducted in the absence of any commercial or financial relationships that could be construed as a potential conflict of interest.

## Publisher's Note

All claims expressed in this article are solely those of the authors and do not necessarily represent those of their affiliated organizations, or those of the publisher, the editors and the reviewers. Any product that may be evaluated in this article, or claim that may be made by its manufacturer, is not guaranteed or endorsed by the publisher.

## References

[1] Xiang Z Si JM Huang HD. Chronic gastritis rat model and role of inducing factors. World J Gastroenterol. (2004) 10:3212–4. 10.3748/wjg.v10.i21.3212 15457578 PMC4611276

[2] Kauer WK Stein HJ. Emerging concepts of bile reflux in the constellation of gastroesophageal reflux disease. J Gastrointest Surg. (2010) 14(suppl. 1):S9–16. 10.1007/s11605-009-1014-4 19756880

[3] Peitz U Wex T Vieth M Stolte M Willich S Labenz J. Correlation of serum pepsinogens and gastrin-17 with atrophic gastritis in gastroesophageal reflux patients: a matched-pairs study. J Gastroenterol Hepatol. (2011) 26:82–9. 10.1111/j.1440-1746.2010.06413.x 21175799

[4] Bennett EJ Kellow JE Cowan H Scott AM Shuter B Langeluddecke PM. Suppression of anger and gastric emptying in patients with functional dyspepsia. Scand J Gastroenterol. (1992) 27:869–74. 10.3109/00365529209000156 1439540

[5] Ladas SD Katsogridakis J Malamou H Giannopoulou H Kesse-Elia M Raptis SA. Helicobacter pylori may induce bile reflux: link between H pylori and bile induced injury to gastric epithelium. Gut. (1996) 38:15–8. 10.1136/gut.38.1.15 8566844 PMC1382972

[6] Lin JK Hu PJ Li CJ Zeng ZR Zhang XG. A study of diagnosis of primary biliary reflux gastritis. Zhonghua Nei Ke Za Zhi. (2003) 42:81–3. 10.3760/j.issn:0578-1426.2003.02.004 12783700

[7] Chen H Li X Ge Z Gao Y Chen X Cui Y. Rabeprazole combined with hydrotalcite is effective for patients with bile reflux gastritis after cholecystectomy. Can J Gastroenterol. (2010) 24:197–201. 10.1155/2010/846353 20352149 PMC2852226

[8] Bhang CS Lee HS Kim SS Song HJ Sung YJ Kim JI. Effects of selective cyclooxygenase-2 inhibitor and non-selective NSAIDs on Helicobacter pylori-induced gastritis in Mongolian gerbils. Helicobacter. (2002) 7:14–21. 10.1046/j.1523-5378.2002.00051.x 11886470

[9] Li D Zhang J Yao WZ Zhang DL Feng CC He Q. The relationship between gastric cancer, its precancerous lesions and bile reflux: A retrospective study. J Dig Dis. (2020) 21:222–9. 10.1111/1751-2980.12858 32187838 PMC7317534

[10] Dixon MF O'Connor HJ Axon AT King RF Johnston D. Reflux gastritis: distinct histopathological entity? J Clin Pathol. (1986) 39:524–30. 10.1136/jcp.39.5.524 3722405 PMC499914

[11] Kellosalo J Alavaikko M Laitinen S. Effect of biliary tract procedures on duodenogastric reflux and the gastric mucosa. Scand J Gastroenterol. (1991) 26:1272–8. 10.3109/00365529108998624 1763297

[12] Atak I Ozdil K Yücel M Caliskan M Kilic A Erdem H. The effect of laparoscopic cholecystectomy on the development of alkaline reflux gastritis and intestinal metaplasia. Hepatogastroenterology. (2012) 59:59–61. 10.5754/hge11244 22260822

[13] Alberti KG Zimmet P Shaw J. The metabolic syndrome–a new worldwide definition. Lancet. (2005) 366:1059–62. 10.1016/S0140-6736(05)67402-8 16182882

[14] Zhang LY Zhang J Li D Liu Y Zhang DL Liu CF. Bile reflux is an independent risk factor for precancerous gastric lesions and gastric cancer: An observational cross-sectional study. J Dig Dis. (2021) 22:282–90. 10.1111/1751-2980.12986 33793080 PMC8252397

[15] Agin M Kayar Y. The effect of primary duodenogastric bile reflux on the presence and density of *Helicobacter pylori* and on gastritis in childhood. Medicina. (2019) 55:775. 10.3390/medicina55120775 31817518 PMC6956137

[16] Egi Y Kim S Ito M Tanaka S Yoshihara M Haruma K. *Helicobacter pylori* infection is the major risk factor for gastric inflammation in the cardia. Dig Dis Sci. (2006) 51:1582–8. 10.1007/s10620-005-9046-4 16602036

[17] Taha AS Angerson WJ Morran CG. Reflux and Barrett's oesophagitis after gastric surgery–long-term follow-up and implications for the roles of gastric acid and bile in oesophagitis. Aliment Pharmacol Ther. (2003) 17:547–52. 10.1046/j.1365-2036.2003.01430.x 12622763

[18] Dixon MF Mapstone NP Neville PM Moayyedi P Axon AT. Bile reflux gastritis and intestinal metaplasia at the cardia. Gut. (2002) 51:351–5. 10.1136/gut.51.3.351 12171955 PMC1773352

[19] Saarinen T Pietiläinen KH Loimaala A Ihalainen T Sammalkorpi H Penttilä A. Bile reflux is a common finding in the gastric pouch after one anastomosis gastric bypass. Obes Surg. (2020) 30:875–81. 10.1007/s11695-019-04353-x 31853864 PMC7347680

[20] Madura JA. Primary bile reflux gastritis: diagnosis and surgical treatment. Am J Surg. (2003) 186:269–73. 10.1016/s0002-9610(03)00213-7 12946831

[21] Shellman Z Aldhahrani A Verdon B Mather M Paleri V Wilson J. Bile acids: a potential role in the pathogenesis of pharyngeal malignancy. Clin Otolaryngol. (2017) 42:969–73. 10.1111/coa.12822 28036160

[22] Yao X Smolka AJ. Gastric parietal cell physiology and helicobacter pylori-induced disease. Gastroenterology. (2019) 156:2158–73. 10.1053/j.gastro.2019.02.036 30831083 PMC6715393

[23] Nakamura M Haruma K Kamada T Mihara M Yoshihara M Imagawa M. Duodenogastric reflux is associated with antral metaplastic gastritis. Gastrointest Endosc. (2001) 53:53–9. 10.1067/mge.2001.111385 11154489

[24] Genta RM. The gastritis connection: prevention and early detection of gastric neoplasms. J Clin Gastroenterol. (2003) 36:S44–9. 10.1097/00004836-200305001-00008 12702965

[25] Siebert M Ribeiro-Parenti L Nguyen ND Hourseau M Duchêne B Humbert L. Long-term consequences of one anastomosis gastric bypass on esogastric mucosa in a preclinical rat model. Sci Rep. (2020) 10:7393. 10.1038/s41598-020-64425-2 32355175 PMC7192900

[26] Li T Guo H Li H Jiang Y Zhuang K Lei C. MicroRNA-92a-1-5p increases CDX2 by targeting FOXD1 in bile acids-induced gastric intestinal metaplasia. Gut. (2019) 68:1751–63. 10.1136/gutjnl-2017-315318 30635407 PMC6839796

[27] Graham DY Osato MS. *H. pylori* in the pathogenesis of duodenal ulcer: interaction between duodenal acid load, bile, and *H. pylori*. Am J Gastroenterol. (2000) 95:87–91. 10.1111/j.1572-0241.2000.01704.x 10638564

[28] Calam J Tracy HJ. Pyloric reflux and G-cell hyperfunction. Lancet. (1980) 2:918. 10.1016/s0140-6736(80)92077-2 6107568

[29] Matsuhisa T Arakawa T Watanabe T Tokutomi T Sakurai K Okamura S. Relation between bile acid reflux into the stomach and the risk of atrophic gastritis and intestinal metaplasia: a multicenter study of 2283 cases. Dig Endosc. (2013) 25:519–25. 10.1111/den.12030 23363381

[30] Tatsugami M Ito M Tanaka S Yoshihara M Matsui H Haruma K. Bile acid promotes intestinal metaplasia and gastric carcinogenesis. Cancer Epidemiol Biomarkers Prev. (2012) 21:2101–7. 10.1158/1055-9965.EPI-12-0730 23010643

[31] Yuan Y Ford AC Khan KJ Gisbert JP Forman D Leontiadis GI. Optimum duration of regimens for Helicobacter pylori eradication. Cochrane Database Syst Rev. (2013) 11:CD008337. 10.1002/14651858.CD008337 24338763 PMC11841770

[32] Fang JY Du YQ Liu WZ Ren JL Li YQ Chen XY. Chinese consensus on chronic gastritis (2017, Shanghai). J Dig Dis. (2018) 19:182–203. 10.1111/1751-2980.12593 29573173

[33] Smolović B Stanisavljević D Golubović M Vucković L Milicić B Djuranović S. Bleeding gastroduodenal ulcers in patients without Helicobacter pylori infection and without exposure to non-steroidal anti-inflammatory drugs. Vojnosanit Pregl. (2014) 71:183–90. 10.2298/vsp1402183s 24665577

[34] Hunt RH Bazzoli F. Review article: should NSAID/low-dose aspirin takers be tested routinely for *H. pylori* infection and treated if positive? Implications for primary risk of ulcer and ulcer relapse after initial healing. Aliment Pharmacol Ther. (2004) 19(suppl. 1):9–16. 10.1111/j.0953-0673.2004.01830.x 14725573

[35] Taha AS Balsitis M Angerson WJ Morran CG. Oesophagitis and bile reflux gastritis–clinical and histological assessments. Dig Liver Dis. (2003) 35:701–5. 10.1016/s1590-8658(03)00410-9 14620618

[36] Bollschweiler E Wolfgarten E Pütz B Gutschow C Hölscher AH. Bile reflux into the stomach and the esophagus for volunteers older than 40 years. Digestion. (2005) 71:65–71. 10.1159/000084521 15775673

[37] Madura JA. Primary bile reflux gastritis: which treatment is better, Roux-en-Y or biliary diversion? Am Surg. (2000) 66:417–23. 10.1097/00000478-200005000-00025 10824740

[38] Luiking YC Akkermans LM Peeters TL Cnossen PJ Nieuwenhuijs VB Vanberge-Henegouwen GP. Effects of motilin on human interdigestive gastrointestinal and gallbladder motility, and involvement of 5HT3 receptors. Neurogastroenterol Motil. (2002) 14:151–9. 10.1046/j.1365-2982.2002.00314.x 11975715

[39] Lu W Sun X Zhang C Wang J Li H Chen H. Effect and mechanism of bile reflux disease on gastric motility induced by benign biliary disease. Chin J Gastroenterol Hepatol. (2009) 18:264–7. 10.3969/j.issn.1006-5709.2009.03.026

[40] Unwin N Shaw J Zimmet P Alberti KG. Impaired glucose tolerance and impaired fasting glycaemia: the current status on definition and intervention. Diabet Med. (2002) 19:708–23. 10.1046/j.1464-5491.2002.00835.x 12207806

[41] Park YW Zhu S Palaniappan L Heshka S Carnethon MR Heymsfield SB. The metabolic syndrome: prevalence and associated risk factor findings in the US population from the Third National Health and Nutrition Examination Survey, 1988-1994. Arch Intern Med. (2003) 163:427–36. 10.1001/archinte.163.4.427 12588201 PMC3146257

[42] Kuk JL Ardern CI. Age and sex differences in the clustering of metabolic syndrome factors: association with mortality risk. Diabetes care. (2010) 33:2457–61. 10.2337/dc10-0942 20699434 PMC2963512

[43] Dallongeville J Cottel D Arveiler D Tauber JP Bingham A Wagner A. The association of metabolic disorders with the metabolic syndrome is different in men and women. Ann Nutr Metab. (2004) 48:43–50. 10.1159/000075304 14646340

[44] Pucci G Alcidi R Tap L Battista F Mattace-Raso F Schillaci G. Sex- and gender-related prevalence, cardiovascular risk and therapeutic approach in metabolic syndrome: A review of the literature. Pharmacol Res. (2017) 120:34–42. 10.1016/j.phrs.2017.03.008 28300617

[45] Vollenweider P von Eckardstein A Widmann C Vollenweider P von Eckardstein A Widmann C. HDLs, diabetes, and metabolic syndrome. Handb Exp Pharmacol. (2015) 224:405–21. 10.1007/978-3-319-09665-0_12 25522996

