# Supplementary material for: Analysis of Risk Factors and Establishment of a Prediction Model for Endoscopic Primary Bile Reflux: A Single-Center Retrospective Study
Source: Front Med (Lausanne). 2021 Nov 10;8:758771. doi: 10.3389/fmed.2021.758771 (PMC8631358; doi:10.3389/fmed.2021.758771)
Supplement: Supplementary file 1 [file Table_1.DOCX]

**Supplementary Table 1** Comparability of baseline characteristics of subjects in the training set and validation set.

| **Characteristic** | **Total (n=844)** | **Training set (n=591)** | **Validation set (n=253)** | **Statistic** |  | ***P–*value** |
| --- | --- | --- | --- | --- | --- | --- |
| Gender, n (%) |  |  |  | *χ*^2^=0.760 |  | 0.383 |
| female | 431 (51.07) | 296 (50.08) | 135 (53.36) |  |  |  |
| male | 413 (48.93) | 295 (49.92) | 118 (46.64) |  |  |  |
| Age, Mean±SD | 49.86±13.93 | 50.18±13.72 | 49.10±14.40 | *t*=-1.03 |  | 0.302 |
| Age, n(%) |  |  |  | *Z*=-0.701 |  | 0.483 |
| <45 | 288 (34.12) | 194 (32.83) | 94 (37.15) |  |  |  |
| 45–59 | 276 (32.70) | 201 (34.01) | 75 (29.64) |  |  |  |
| ≥60 | 280 (33.18) | 196 (33.16) | 84 (33.20) |  |  |  |
| Height, M(Q1,Q3) | 1.65(1.60,1.72) | 1.65(1.60,1.72) | 1.65(1.60,1.72) | *Z*=-0.838 |  | 0.402 |
| Weight, Mean±SD | 64.26±13.73 | 64.23±13.45 | 64.35±14.39 | *t*=0.12 |  | 0.906 |
| BMI, Mean±SD | 23.19±3.95 | 23.14±3.87 | 23.31±4.14 | *t*=0.56 |  | 0.577 |
| BMI, n (%) |  |  |  | *χ*^2^=2.005 |  | 0.917 |
| ＜18.5 | 84(9.95) | 59(9.98) | 25(9.88) |  |  |  |
| 18.5–23.9 | 430(50.95) | 300(50.76) | 130(51.38) |  |  |  |
| ≥24 | 330(39.10) | 232(39.26) | 98(38.74) |  |  |  |
| NSAIDs use history, n (%) |  |  |  | *χ*^2^=2.006 |  | 0.157 |
| No | 711 (84.24) | 491 (83.08) | 220 (86.96) |  |  |  |
| Yes | 133 (15.76) | 100 (16.92) | 33 (13.04) |  |  |  |
| Chronic liver diseases, n(%) |  |  |  | *χ*^2^=0.238 |  | 0.626 |
| No | 741 (87.80) | 521 (88.16) | 220 (86.96) |  |  |  |
| Yes | 103 (12.20) | 70 (11.84) | 33 (13.04) |  |  |  |
| Cholelithiasis, n(%) |  |  |  | *χ*^2^=0.002 |  | 0.965 |
| No | 638 (75.59) | 447 (75.63) | 191 (75.49) |  |  |  |
| Yes | 206 (24.41) | 144 (24.37) | 62 (24.51) |  |  |  |
| *H.pylori* infection, n(%) |  |  |  | *χ*^2^=0.251 |  | 0.616 |
| No | 641 (75.95) | 446 (75.47) | 195 (77.08) |  |  |  |
| Yes | 203 (24.05) | 145 (24.53) | 58 (22.92) |  |  |  |
| Psychological factors, n(%) |  |  |  | *χ*^2^=0.023 |  | 0.879 |
| No | 584 (69.19) | 408 (69.04) | 176 (69.57) |  |  |  |
| Yes | 260 (30.81) | 183 (30.96) | 77 (30.43) |  |  |  |
| Allergic constitution, n(%) |  |  |  | *χ*^2^=0.757 |  | 0.384 |
| No | 682 (80.81) | 473 (80.03) | 209 (82.61) |  |  |  |
| Yes | 162 (19.19) | 118 (19.97) | 44 (17.39) |  |  |  |
| Erosive gastritis, n(%) |  |  |  | *χ*^2^=0.008 |  | 0.928 |
| No | 602 (71.33) | 421 (71.24) | 181 (71.54) |  |  |  |
| Yes | 242 (28.67) | 170 (28.76) | 72 (28.46) |  |  |  |
| Smoking, n(%) |  |  |  | *χ*^2^=0.231 |  | 0.631 |
| No | 679 (80.45) | 478 (80.88) | 201 (79.45) |  |  |  |
| Yes | 165 (19.55) | 113 (19.12) | 52 (20.55) |  |  |  |
| Alcohol consumption, n(%) |  |  |  | *χ*^2^=0.003 |  | 0.956 |
| No | 758 (89.81) | 531 (89.85) | 227 (89.72) |  |  |  |
| Yes | 86 (10.19) | 60 (10.15) | 26 (10.28) |  |  |  |
| metabolic syndrome, n(%) |  |  |  | *χ*^2^=1.838 |  | 0.175 |
| No | 646 (76.54) | 460 (77.83) | 186 (73.52) |  |  |  |
| Yes | 198 (23.46) | 131 (22.17) | 67 (26.48) |  |  |  |
| Pancreatic diseases, n(%) |  |  |  | *χ*^2^=2.865 |  | 0.091 |
| No | 809 (95.85) | 562 (95.09) | 247 (97.63) |  |  |  |
| Yes | 35 (4.15) | 29 (4.91) | 6 (2.37) |  |  |  |
| Gastrointestinal symptoms,n(%)  No  Yes | 486(57.58)  358(42.42) | 342(57.87)  249(42.13) | 144(56.92)  109(43.08) | *χ*^2^=0.066 |  | 0.798 |
| Bile reflux, n(%) |  |  |  | *χ*^2^=0.000 |  | 0.992 |
| No | 280 (33.18) | 196 (33.16) | 84 (33.20) |  |  |  |
| Yes | 564 (66.82) | 395 (66.84) | 169 (66.80) |  |  |  |
